# PBJ is ranked higher, publishes more original articles and offers free global access

**DOI:** 10.1111/pbi.13069

**Published:** 2019-01-13

**Authors:** Henry Daniell

**Affiliations:** ^1^ Director of Translational Research University of Pennsylvania Philadelphia PA USA

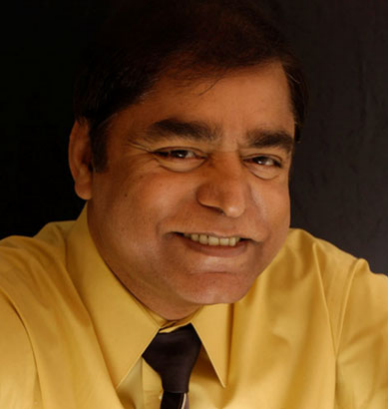

Welcome to the first issue of the seventeenth volume of the *Plant Biotechnology Journal*. *PBJ* has successfully transitioned from a subscription based journal to an open access journal, ranked by Scopus as second among 238 open access journals published in all fields of research and is the only open access journal among the top ten plant science journals. This transition has resulted in enhanced free global access to all our readers.

Scopus CiteScore also ranks *PBJ* first among 309 Agronomy and Crop Science journals. Excluding review journals in plant sciences, CiteScore ranks *PBJ* third (6.51) behind *Plant Cell* (7.33) and *New Phytologist* (6.65). Both *Plant Journal and Plant Physiology* (6.01) are ranked fourth and *Molecular Plant* and *Nature Plants* (5.71) are ranked seventh. CiteScore metrics addresses some of the major limitations of the ISI impact Factor, which excludes certain article types (Letters, Commentaries, etc) thereby decreasing the number of citable items counted in the denominator of the impact factor. For example, *Nature Plants* (11.47) and *Molecular Plant* (9.32) are ranked highest by impact factor score amongst plant science journals publishing original research but are ranked much lower among the top ten by the CiteScore. Therefore, most journals including *PBJ* now list both scores on their websites to highlight such discrepancies in journal impact. *PBJ* ranks third based on CiteScore and fifth based on impact factor among plant science journals publishing original research. Irrespective of the method of citation analysis, *PBJ* is building on the strength of high impact original inventions reported by our authors and critically evaluated by our editors/reviewers. Therefore, I convey my greatest appreciation to our authors for submitting their best research and our editors/reviewers for their critical evaluations. I look forward to your continued support in 2019.

While it is relatively easy to reduce the number of published articles in order to improve journal ranking, it is quite challenging to publish high quality original research and also meet the publication needs of the plant research community. As the Editor in Chief of *PBJ*, I have accepted this challenge and continue to encourage publication of more research articles. Therefore, *PBJ* received a record number of manuscripts in 2018 from all continents, with more submissions from Asia (114%), Middle East (250%), Central /South America (163%), North America (111%) and Oceania (104%) but a slight (−6%) decrease from Europe. Of course, this has increased required the number of *PBJ* reviewers and I am eternally grateful for their critical evaluations and generous time commitment. I am happy to report that the increase in number of reviewers didn't significantly increase the average turnaround time, rewarding our authors with timely decisions on their submissions. Considering *PBJ* is still a very young journal, these are very impressive accomplishments.

*PBJ* Altmetric scores of high impact articles are, at times, higher than those published in *Science* or *Nature* journals. The *PBJ* website has highlighted the top three articles with very high Altmetric scores on polio booster vaccine (558), low‐gluten non‐transgenic wheat (509) and golden bananas (242). In order to further engage the public or policy makers in scientific discussions, I request that authors provide *Twitter titles* at the time of manuscript submission. In addition, I encourage authors to share news releases on their articles with the *PBJ* editorial office so that they are included in Wiley Plant Science tweets @PlantSciNews, which currently has ~13 500 followers.

As an applied plant biology journal, *PBJ* has a unique role in building bridges between academia, industry and regulatory agencies and also in increasing awareness of plant biotechnology research among the public. While full length articles encompassing in depth studies meet scientific needs, they are not suitable when engaging the public and regulatory agencies. Therefore, I encourage authors to submit “*PBJ* letters” to briefly report new inventions or commentaries (limit: three printed page articles, <1500 words, ten citations and one illustration, no supplementary files). *PBJ* will not publish more letters or commentaries to manipulate journal ranking and will restrict this for it's intended purpose. Letters submitted to *PBJ* will also undergo the same rigorous peer review that we expect of original research articles.

With the support of *PBJ* management, I have significantly increased the number of associate editors, representing several new areas and different continents, not previously represented. We welcome new associate editors: Dr. François Belzile, Department of Plant Sciences, Institute for Integrative and Systems Biology, Laval University, Québec, Canada; Dr. Shuangxia Jin, National Key Laboratory of Crop Genetic Improvement, Huazhong Agricultural University, China; Dr. Jihong Liu‐Clarke, NIBIO, Norwegian Institute of Bioeconomy Research, Norway and Dr. Marco Maccaferri, Department of Agricultural Science, University of Bologna, Bologna, Italy. The *PBJ* editorial board has also been revitalized by replacing less active members with investigators in emerging new disciplines.

I convey my sincere thanks for the excellent service offered by all our Associate Editors who have served more than 10 years (Profs. Dominique Michaud, Malcolm Campbell) or in the past few years (Profs. Neal Stewart, Dave Edwards, Stephen Streatfield, Johnathan Napier, Xiao‐Ya Chen, Nicola Patron, Martin Parry, Rajeev Varshney, Kan Wang) or joined recently (Drs. Caixia Gao, Zuhua He), and also the Editorial staff (Jim Ruddock – Managing Editor, Rosie Trice‐ Senior Publishing Manager, Cathryn Jordan‐Editorial Assistant, Hannah Qualtrough‐Senior Marketing Manager) at Wiley, Oxford. I thank Julie Ann Suliguin, who served as *PBJ* production editor for many years but moved on to another position recently, and I welcome our new production editor Ms. Maricar Dumlao, Wiley, Manila.

*PBJ* is now compatible with mobile platforms, tablets, iPads and iPhones and offers several new options to evaluate the short and long‐term impact of published articles, including Altmetric scores, article readership and citations. I encourage all readers to visit the journal homepage to take advantage of open access, keep up to date with latest developments and to sign up for our automated e‐alerts in order to receive emailed notifications when new issues or Early View articles are published. Please note that readers should ‘opt‐in’ to receive e‐alerts, by visiting the journal homepage and registering at the ‘Get Content Alerts’ area.

*PBJ* management has approved my request to waive or reduce open access fees for manuscripts recommended for publication from authors who do not have adequate funding for publications. Therefore, I am fully committed to advancing *PBJ*'s mission of publishing high quality manuscripts with free global access and look forward to your continued support in 2019.

